# Mechanistic insight into human milk extracellular vesicle‐intestinal barrier interactions

**DOI:** 10.1002/jex2.70032

**Published:** 2025-01-09

**Authors:** Xiang Luo, Yunyue Zhang, Ning Ding, Jana Javorovic, Bahijja Tolulope Raimi‐Abraham, Steven Lynham, Xiaoping Yang, Natalie Shenker, Driton Vllasaliu

**Affiliations:** ^1^ Institute of Pharmaceutical Science School of Cancer and Pharmaceutical Science King's College London London UK; ^2^ Centre of Excellence for Mass Spectrometry, The James Black Centre King's College London London UK; ^3^ Institute of Reproductive and Developmental Biology Imperial College London UK

**Keywords:** EV cell uptake, EV intestinal translocation, EV proteomics, human milk EVs

## Abstract

Human milk extracellular vesicles (EVs) are crucial mother‐to‐baby messengers that transfer biological signals. These EVs are reported to survive digestion and transport across the intestine. The mechanisms of interaction between human milk EVs and the intestinal mucosa, including epithelial uptake remain unclear. Here, we studied the interaction of human milk EVs with the gut barrier components, including intestinal biofluids, enzymes, mucus and epithelium. Additionally, we probed the endocytic mechanisms mediating the EV intestinal uptake. Finally, using proteomic analysis, we determined the existence and identification of proteins enriched in the EV fraction transported across the intestinal epithelium. We show that human milk EVs are largely stable in the biochemical gut barriers and demonstrate high mucus diffusivity. EVs show a high level of epithelial cell uptake (∼70%) and efficient transport across Caco‐2 monolayers. Whilst cell uptake of EVs was mediated by multiple routes, none of the pathway‐specific inhibitors inhibited their epithelial translocation. Proteomic analysis of EVs transported across Caco‐2 monolayers identified 14 enriched EV proteins that may facilitate intestinal transport. These findings significantly expand our understanding of the interactions between human milk EVs and the gut barriers, including their intestinal uptake.

## 1 INTRODUCTION

Extracellular vesicles (EVs; also referred to as exosomes—a subtype of EVs) are lipid bilayer‐enclosed particles secreted from cells into the extracellular space. EVs encapsulate biological molecules including proteins, lipids and genetic material, protecting them against enzymatic degradation and transporting them between cells (Colombo et al., 2014). EVs are found in various biological fluids including milk, into which they are secreted by mammary gland cells such as mammary epithelial cells, immune cells, stem cells and adipocytes (Sanwlani et al., 2020). They are heterogenous in size, spanning 30−150 nm for small EVs (Sanwlani et al., 2020), and are present in human milk at a density of 10^9^−10^10^ particles per mL (Vaswani et al., 2019). Human milk EVs are thought to play a critical role in the mother‐to‐baby transfer of important biological signals (Jiang et al., 2021; Kupsco et al., 2021). These EVs may therefore play a key role in imparting the health benefits of human milk to the baby, although currently this field that is not well understood.

Key to the role of human milk EVs as messengers of biological signals to the baby is their reported capability to survive digestion and transport across the human intestinal epithelium (Jiang et al., 2021). Studying human milk EVs is also interesting from a drug delivery perspective because there is an unmet need for drug delivery vectors that possess such criteria, with existing synthetic drug delivery systems (e.g., liposomes) lacking stability in the gut environment or being unable to efficiently permeate the key physical barriers of the intestinal mucosa (i.e., mucus and epithelium). In this work, we studied the interactions of human milk small EVs (of diameter of 150 nm or below) with the key biological barriers of the human intestine, namely intestinal fluids, gut enzymes, mucus and epithelium. Furthermore, we derived mechanistic insight in terms of the intestinal epithelial transport processes involved in the gut uptake of these EVs. Finally, we employed proteomic analysis to determine whether the fraction of EVs transported across the intestinal epithelium is enriched in specific proteins, which may play a role in facilitating this transport. The results confirm the previous reports regarding the stability and intestinal transport of human milk EVs and offer new insight into mucus diffusivity, endocytic mechanisms of epithelial uptake, and the composition of human milk EV subpopulations transported across the intestinal epithelium. This work provides novel mechanistic inference into the biological understanding of the intestinal absorption of human milk EVs.

## 2 METHODS

### 2.1 Materials

Human milk was kindly donated by the Hearts Milk Bank (Hertfordshire, UK). Dulbecco's modified Eagle's medium (DMEM), a QuantiPro BCA Assay Kit, Triton X‐100, sodium dodecyl sulphate (SDS), N,N‐dimethylformamide, Hank's balanced salt solution (HBSS), foetal bovine serum (FBS, non‐USA origin), non‐essential amino acids, antibiotic/antimycotic solution, Fluoroshield DAPI, low‐temperature gelling agarose and paraformaldehyde were obtained from Merck (Dorset, UK). Sodium acetate, hydrochloric acid and acetic acid were purchased from Sigma‐Aldrich (St. Louis, USA). Fed‐ and Fasted‐State Simulated Intestinal Fluids (FeSSIF and FaSSIF, respectively) were purchased from Biorelevant (London, UK). α‐Amylase was purchased from MedChemExpress (South Brunswick, NJ, USA). Pepsin was bought from Promega (Madison, WI, USA). Pancreatin was purchased from TargetMol (Boston, MA, USA). Lypase‐A was bought from Antibodies.com (Cambridge, UK). 1,6‐Diphenyl‐1,3,5‐hexatriene (DPH) was purchased from Cayman Chemical (Ann Arbor, MI, USA). Ambion Nuclease‐Free Water, TRIzol reagent, ZO‐1 polyclonal antibody and chicken anti‐rabbit IgG (H+L) cross‐adsorbed secondary antibody conjugated with Alexa Fluor 488 and DiD dye were bought from Thermo Fisher Scientific (Waltham, MA, US). Caco‐2 cells were purchased from the European Collection of Cell Cultures (ECACC, Salisbury, UK) and used between passage p15−35. An ExoGlow‐Protein EV Labeling Kit (Red), ExoQuick reagent and an Exo‐Check Exosome Antibody Array kit were purchased from System Biosciences (Palo Alto, CA, USA). 12 mm Transwells with 0.4 µm pore polycarbonate membrane inserts were purchased from Corning (Glendale, AZ, USA). Vybrant DiD solution, Fluospheres, and fluorescently labelled (red) carboxyl‐modified polystyrene latex nanoparticles (100 and 200 nm diameter) were purchased from Thermo Fisher Scientific (Waltham, MA, US). Bovine pasteurised skimmed milk was purchased from a local grocery (Sainsbury's, London, UK). Ibidi chambered coverslips were acquired from Thistle Scientific (Rugby, UK). Porcine stomachs were sourced from Cheale Meats (Essex, UK) from animals slaughtered for commercial use. Sodium chloride (ACS reagent ≥99.0%), ethylene diamine tetra acetic acid (EDTA; ACS reagent 99.4%−100.6%), sodium azide (ReagentPlus ≥99.5%), and phenylmethylsulphonyl fluoride (PMSF, ≥99.0%) were obtained from Sigma‐Aldrich (Dorset, UK). Visking dialysis tubing (MWCO 12−14 kDa) was purchased from Fisher Scientific (Loughborough, UK).

### 2.2 EV isolation and characterisation

EVs were isolated from human milk by a pH adjustment and differential ultracentrifugation process optimised based on previous studies (Zhang et al., 2023). Briefly, milk pH (6.5) was adjusted to 4.6 using pH 4 acetate buffer containing 1.02 g sodium acetate and 4 mL acetic acid dissolved in 50 mL phosphate‐buffered saline (PBS). Milk (70 mL) was centrifuged at 13,000 × *g* for 30 min at 4°C and the supernatant was transferred to new centrifuge tubes to remove fats and proteins (whey and casein). Whey was then centrifuged at 100,000 × *g* for 60 min at 4°C to pellet large particles. The supernatant was filtered using 0.2 µm filters to remove large particles and further centrifuged at 135,000 × *g* for 90 min at 4°C with an Optima XPN‐80 Ultracentrifuge (Type 45 Ti fixed angle rotor; Beckman Coulter, Brea, CA, USA), producing an EV pellet that was washed with 70 mL of PBS and centrifuged at 135,000 × *g* for 90 min. The pellet was resuspended in 1 mL sterile PBS. Purified EVs in sterile PBS were stored at −80°C for up to 3 months. EVs from different donors were stored and studied separately (as biological replicates) downstream.

#### 2.2.1 Determination of EV protein concentration

The total protein concentration of EVs was determined using a QuantiPro BCA Assay Kit following the manufacturer's protocol. The absorbance of triplicate samples was measured at 562 nm using an Infinite 200 Pro plate reader (Tecan, Männedorf, Switzerland). Surface charge (zeta‐potential) was measured with a Malvern Zetasizer (Malvern, UK). Expression of exosome protein markers was determined using an Exo‐Check Exosome Antibody Array Kit following the manufacturer's instructions.

#### 2.2.2 Nanoparticle tracking analysis (NTA)

NTA was employed to measure the size distribution and particle concentration of EVs using an LM10 Particle sizer (Nanosight, Malvern, UK). Samples were diluted at 1:1000 to achieve a particle concentration in the range of 10^8^ to 10^9^ particles/mL. Three captures of 30 s for each sample were recorded and data were analysed using NanoSight NTA software. A red laser with a wavelength of 642 nm was used together with a camera level of 8−12 and detection threshold of 2, which ensured that the particle per frame value was within 20−100 particles/frame.

#### 2.2.3 EV imaging

EVs were imaged with a JEM 1400 Flash transmission electron microscope (JEOL, Tokyo, Japan) at 120 kV. Before use, 300 mesh copper grids were glow‐discharged using a GloQube Plus Discharge system (Quorum, Newcastle upon Tyne, UK). The 3 µL samples were applied on the carbon side of the grids for 30 s, followed by staining of the grids in 30 µL of 3 % uranyl acetate for 2 min.

### 2.3 In vitro digestion of EVs

FaSSIF (pH 6.5) and FeSSIF (pH 5.0) were used as cost‐effective, commercially available models of small intestinal fluids with bile salts. Simulated small intestinal fluids (SSIFs) were prepared according to the manufacturer's instructions. Intestinal digestion was simulated by incubating 100 µL of EV suspension at 1 mg/mL protein concentration in 900 µL of SSIFs or control (1% SDS) at 37°C with light shaking for 1.5 h. After digestion, EVs were recovered via centrifugal ultrafiltration with 100 kDa Amicon centrifugal filter units (Merck, Darmstadt, Germany) via four sequential centrifugations for 10 min each, with PBS (500 µL) addition between each centrifugation step to wash the digestion solution from the pellet. Finally, EVs were resuspended in 200 µL PBS and their size was determined using NTA and dynamic light scattering (DLS).

To investigate the stability of EVs under digestive conditions, 100 µL of 4 mg/mL EVs was treated with 300 µL of individual enzyme solutions at 37°C with light shaking for 1.5 h. α‐Amylase (3 µg/mL) in ultrapure water was used to simulate the salivary environment (Avila‐Sierra et al., 2024). To simulate gastric fluids, pepsin was prepared at 10 µg/mL in 10 mM HCl at pH 1.5. For intestinal stability studies, EVs were incubated with 30 µg/mL pancreatin in FaSSIF. EVs were also treated with lipase at 10 µg/mL. Digestive enzymes were inactivated by exposure to ice‐cold conditions after incubation, followed by ultrafiltration to remove enzymes and EV debris (if any). The size of EVs was determined as described above following treatment.

### 2.4 EV membrane stability following in vitro digestion

To determine the membrane stability of EVs following in vitro digestion we used DPH as a fluorescent probe, which is highly fluorescent when incorporated in a lipid environment (such as the membrane bilayer of EVs), but is associated with a significant loss of fluorescence signal when present in an aqueous environment (Zhang et al., 2024). DPH was dissolved in N,N‐dimethylformamide to form a stock solution. EVs were diluted to a concentration of 0.56 mg/mL and stained with 2.3 µM DPH by incubating for 40 min at room temperature under gentle agitation in the dark. SSIFs were prepared as described above, and 100 µL of DPH‐EV suspensions were exposed to SSIFs for 1.5 h in the dark at 37°C under gentle agitation. Thereafter, DPH‐stained EVs were recovered by ultrafiltration and resuspended in PBS as described above. The fluorescence signal of the recovered EVs was measured by an Infinite 200 Pro plate reader at excitation and emission wavelengths of 360 and 430 nm, respectively. Control experiments were carried out under similar conditions, with the replacement of SSIFs by PBS (negative control) and SDS (1% w/v) as a known membrane‐disrupting agent.

### 2.5 Single‐particle tracking

For particle tracking analysis, human milk EVs were labelled with DiD dye. Briefly, 1.5 µL of DiD dye was incubated with 500 µL of EVs at 37°C for 20 min with shaking. Next, 167 µL of ExoQuick reagent was added and incubated at 4°C overnight to precipitate EVs. EVs were quantified by a bicinchoninic acid (BCA) assay and size was determined by DLS.

Native porcine gastric mucus was prepared according to previously published protocols (Chen et al., 2019; Trenkel & Scherließ, 2021). Briefly, porcine gastric mucus was acquired by scraping the inner lining of the stomach of slaughtered pigs. The acquired crude mucus was mixed 1:1 with protease‐inhibiting buffer containing 200 mM sodium chloride, 5 mM EDTA, 0.02% (w/v) sodium azide and 1 mM phenylmethylsulphonyl fluoride. The mixture was purified by centrifugation using an Optima XPN‐80 ultracentrifuge (Beckman Coulter) at 11,200 × *g* for 45 min at 4°C and dialysed against distilled water using Visking dialysis tubing. The solution was then concentrated using an Amicon ultra‐filtration stirred cell under nitrogen at 40 psi. The purified mucus was dried to a constant weight and reconstituted with buffer to a concentration of 3% (w/v).

Polystyrene latex nanoparticles of 100 nm (PL100) and 200 nm (PL200) diameter were pre‐diluted to a concentration of 5.4 × 10^10^ particles/mL in ultrapure water (18.2 MΩ·cm). DiD‐labelled EVs were pre‐diluted to a concentration of 1.0 × 10^11^ particles/mL in PBS. Nanoparticle suspensions (5 µL) were added to native porcine gastric mucus (200 µL; 3% [w/v]) with gentle mixing for 10 s before being transferred onto a multichannel coverslip for imaging. Nanoparticles dispersed in mucus were imaged using a Nikon Ti2 inverted microscope (Nikon Instruments, Amstelveen, The Netherlands) equipped with a Hamamatsu Orca Flash 4.0 camera (Hamamatsu Photonics, Herrsching am Ammersee, Germany) using a CFI SR HP Plan Apochromat Lambda S 100XC Sil objective (Nikon Instruments) while maintaining a constant temperature of 25°C. Images were collected over a 20 s duration at a rate of 50 frames/s.

Images were analysed using ImageJ software with the TrackMate plugin (Tinevez et al., 2017). Approximately 360−390 individual tracks were collected per sample with generated tracks subsequently exported into MATLAB and analysed using a publicly available package developed for mean squared displacement (MSD) analysis of particle trajectories (Tarantino et al., 2014). The diffusion coefficient *D* of each type of particle was determined using least‐square fit of time‐averaged MSD values of individual particle tracks. To assess whether particles are confined or freely diffusing within the medium, the slope of the log‐log fit was determined from individual particle tracks, discarding MSD curves with *R* ^2^ < 0.7 (Tarantino et al., 2014).

### 2.6 Intestinal epithelial cell uptake of EVs

#### 2.6.1 Flow cytometry

Caco‐2 cells were seeded at 120,000 cells/well in 12‐well plates and incubated for 48 h. Human milk EVs were labelled with DiD dye and characterised as described above. Caco‐2 cells were initially equilibrated in HBSS for 45 min before incubation with EVs for 3 h at 37°C and a 5% CO_2_ atmosphere. After treatment, cells were washed three times in PBS and detached with trypsin (10 min incubation). PBS (500 µL) with 3% v/v FBS and 5 mM EDTA was added to neutralise the reaction, followed by centrifugation for 10 min to collect the cell pellet. For cell fixation, pellets were resuspended and incubated in 100 µL 4% paraformaldehyde for 20 min at room temperature. Cells were collected by centrifugation and 30 µL of DAPI was added to label nuclei. Finally, PBS was added and cells were stored on ice in the dark until analysis. All fluorescence‐activated cell sorting data were collected from DAPI and Allophycocyanin (APC) channels on a CytoFlex LX flow cytometer (Beckman Coulter) and analysed by CytExpert (Beckman Coulter).

#### 2.6.2 Confocal microscopy

Differentiated Caco‐2 cells cultured on Transwell inserts for 21−23 days were incubated with EVs at 0.05 mg/mL for 3 h. EVs were removed, cells were washed with PBS, and fixed with 4% paraformaldehyde as described above. Cells were permeabilised with 0.2% (v/v) Triton X‐100 for 5 min and blocked with 5% skimmed milk containing 0.05% (v/v) Triton X‐100 for 30 min. Cellular tight junctions were stained with ZO‐1 primary antibody (1:150 dilution) and IgG (H+L) secondary antibody conjugated with Alexa Fluor 488 (1:500 dilution). Cell nuclei were stained with DAPI. Samples were embedded on Ibidi chambered coverslips and imaged on an Eclipse Ti Inverted Spinning Disk confocal microscope (Nikon Instruments).

### 2.7 Transport of EVs across differentiated Caco‐2 monolayers

Caco‐2 cells were cultured on 12‐well polycarbonate Transwell inserts for 21−23 days with regular exchange of culture medium and measurement of transepithelial electrical resistance (TEER) to ensure monolayer barrier integrity, only cell monolayers with TEER > 1000 Ω/cm^2^ were used in experiments.

EVs were labelled using an ExoGlow EV protein labelling kit (Red) as described above. Before the transport study, Caco‐2 cells were equilibrated with HBSS for 45 min at 37°C and 5% CO_2_ atmosphere. Thereafter, 500 µL of labelled EVs at a protein concentration of 0.05 mg/mL in pre‐warmed HBSS were added to the apical side of monolayers and incubated for 3 h. During the incubation, 100 µL of basolateral solution was sampled regularly (at 30 min intervals) and the sampled solution was replaced with HBSS. EVs in the sampled basolateral solution were quantified by fluorescence using a plate reader with excitation and emission wavelengths of 565 and 615 nm, respectively.

To test whether enzymatic treatment of EVs had an impact on their ability to translocate across the intestinal epithelium, DID‐labelled EVs were exposed to enzymatic treatment as described in Section 2.3, then tested for intestinal epithelial transport in Caco‐2 monolayers as described above and 3 h samples were collected to measure.

### 2.8 Determination of endocytic pathways of EV uptake and transport

Well‐established pharmacological inhibitors of endocytic pathways were used to determine the relative contributions of specific pathways in intestinal epithelial uptake and transport of EVs. The concentrations of these inhibitors were previously optimised by Bannunah et al. (Bannunah et al., 2014), including chlorpromazine (20 µg/mL), genistein (50 µg/mL), nocodazole (12.5 µg/mL), 5‐(N‐ethyl‐N‐isopropyi)‐amiloride (EIPA) (10 µg/mL), methyl‐β‐cyclodextrin (6650 µg/mL) and dynasore (20 µg/mL) in HBSS. Cell monolayers were incubated with these inhibitors for 30 min at 37°C in a 5% CO2 atmosphere. The inhibitors were removed and replaced with EVs in HBSS (0.05 mg/mL) containing each inhibitor. EVs (0.05 mg/mL) served as controls (no inhibition). Cell uptake and permeability of EVs in the presence of these inhibitors were measured as described above using flow cytometry and fluorescence‐based quantitation of periodically sampled basolateral solutions for uptake and transport, respectively.

### 2.9 Proteomic analysis of EVs

To probe the mechanism of intestinal epithelial translocation of human milk EVs, specifically to identify any EV proteins that may facilitate transport, we used quantitative proteomics to analyse EVs following intestinal transport. To this end, EVs applied to intestinal Caco‐2 monolayers were analysed by liquid chromatography‐mass spectrometry (LC‐MS) following transport, with a comparison made between EVs remaining on the apical side and those translocating to the basolateral. EVs on both sides were collected and concentrated to 10 µL in PBS using 100 kDa centrifugal filter units. Proteins were purified by gel electrophoresis to remove lipids and sugar compounds. MS samples were prepared and analysed by King's College London proteomic facility. The EVs protein gel bands were tryptic digested by following routine in‐gel digestion protocol. Briefly, the gel bands were destained and tryptic digested after being treated with dithiothreitol (DTT) and iodoacetamide (IAA), the digested peptides were extracted from gel pieces with acetonitrile and speedVac dried before LC‐MS analysis.

The tryptic peptides were subjected to an LC‐MS system for analysis. For liquid chromatography, a reverse phase Thermo Acclaim Pepmap trap column (2 cm in length, 75 µm in diameter and 3 µm C18 beads) was connected to a nanoflow HPLC (RSLC Ultimate 3000) on an Easy‐spray C18 nano column (50 cm length, 75 µm in diameter, Thermo Fisher Scientific, US). Buffer A (5% ACN, 0.1% formic acid) and buffer B (80% ACN, 0.1% formic acid) were used. Peptides were eluted with a linear gradient of 5%–55% buffer B at a flow rate of 250 nL/min over 60 min at 45°C. Peptides were directly ionized within the easyspray ion source (Thermo, US) and injected into Orbitrap Fusion Lumos mass spectrometers (Thermo, US).

MS data generated were collected within Xcalibur 4.4 to acquire MS data using a ‘Universal’ method by defining a 3s cycle time between a full MS scan and MS/MS fragmentation. We acquired one full‐scan MS spectrum at a resolution of 120,000 at 200 m/z with a normalized automatic gain control (AGC) target (%) of 250 and a scan range of 300–1600 m/z. The MS/MS fragmentation was conducted using CID collision energy (35%) with an orbitrap resolution of 30,000 at 200 m/z. The AGC target (%) was set up as 200 with a max injection time of 128 ms. A dynamic exclusion of the 30s and 2–7 included charged states were defined within this method.

The resulting raw files were searched against the human database downloaded from Uniprot/Swiss‐Port (9 March 2024) within Thermo Proteome Discoverer (PD, version 2.5) allowing two missed cleavage sites and methionine oxidation. Carbamidomethylation on cysteine residues was set as a fixed modification. Precursor mass tolerance was set as 20 ppm and fragment ion tolerance was set as 0.6 Da. PSMs that met the false discovery rate cut‐off of 1% based on the search against a decoy database were considered for further analysis. MS identification included proteins identified with only one peptide.

To identify EV proteins that may mediate epithelial translocation, the profiles of EV samples in the apical and basolateral chambers were compared. Precursor ion intensities extracted from PD were applied for differential analysis using a two‐sample *t*‐test algorithm or analysis of variance (ANOVA) algorithm embedded in Perseus software. Differences with *p* < 0.05 were considered significant. *, **, *** and **** indicate *p* < 0.05, *p* < 0.01, *p* < 0.001 and *p* < 0.0001, respectively. Results are presented as the mean ± standard deviation (SD) from at least three technical replicates and biological replicates. Proteins identified in at least three out of five replicates (or above 70%) were considered as positive identification. Fold changes at the protein level between apical and basolateral chambers were calculated using quantitative ion intensities.

Protein–protein interaction network (PPI) analysis of EVs from donor 1 was carried out using the STRING database.

### 2.10 Statistical analysis

Data were analysed using GraphPad Prism (GraphPad Software Inc, La Jolla, CA, USA). Results are presented as the mean ± SD from at least three technical replicates and biological replicates. Statistical analysis was performed by unpaired Student's *t*‐test or analysis of variance (ANOVA). Differences with *p* < 0.05 were considered significant. *, **, *** and **** indicate *p* < 0.05, *p* < 0.01, *p* < 0.001 and *p* < 0.0001, respectively.

## 3 RESULTS

### 3.1 EV isolation and characterisation

Table 1 shows the size (123 nm−131 nm) and zeta‐potential (slightly negatively charged) of isolated human milk EVs. These fall within the range of values reported previously (Bickmore & Miklavcic, 2020).

**TABLE 1:** Physicochemical parameters of human milk EVs.

Parameter	Measurement method	Value
Size (nm)	DLS^a^	130.9 ± 1.8
Size (nm)	NTA^b^	123.4 ± 10.3
PdI^c^	DLS	0.288
Zeta‐potential (mV)	DLS	−5.56 ± 0.90
Particle concentration (particles/mL of human milk)	NTA	(3.4 ± 0.6) × 10^9^
Protein concentration (mg/mL of human milk)	BCA^d^	0.14

*Note*: The results are the mean ± SD (*n* = 3).

Abbreviation: EV, extracellular vesicles.

^a^ DLS, dynamic light scattering.

^b^ NTA: nanoparticle tracking analysis.

^c^ PdI, polydispersity index.

^d^ BCA: bicinchoninic acid assay.

**FIGURE 1:**
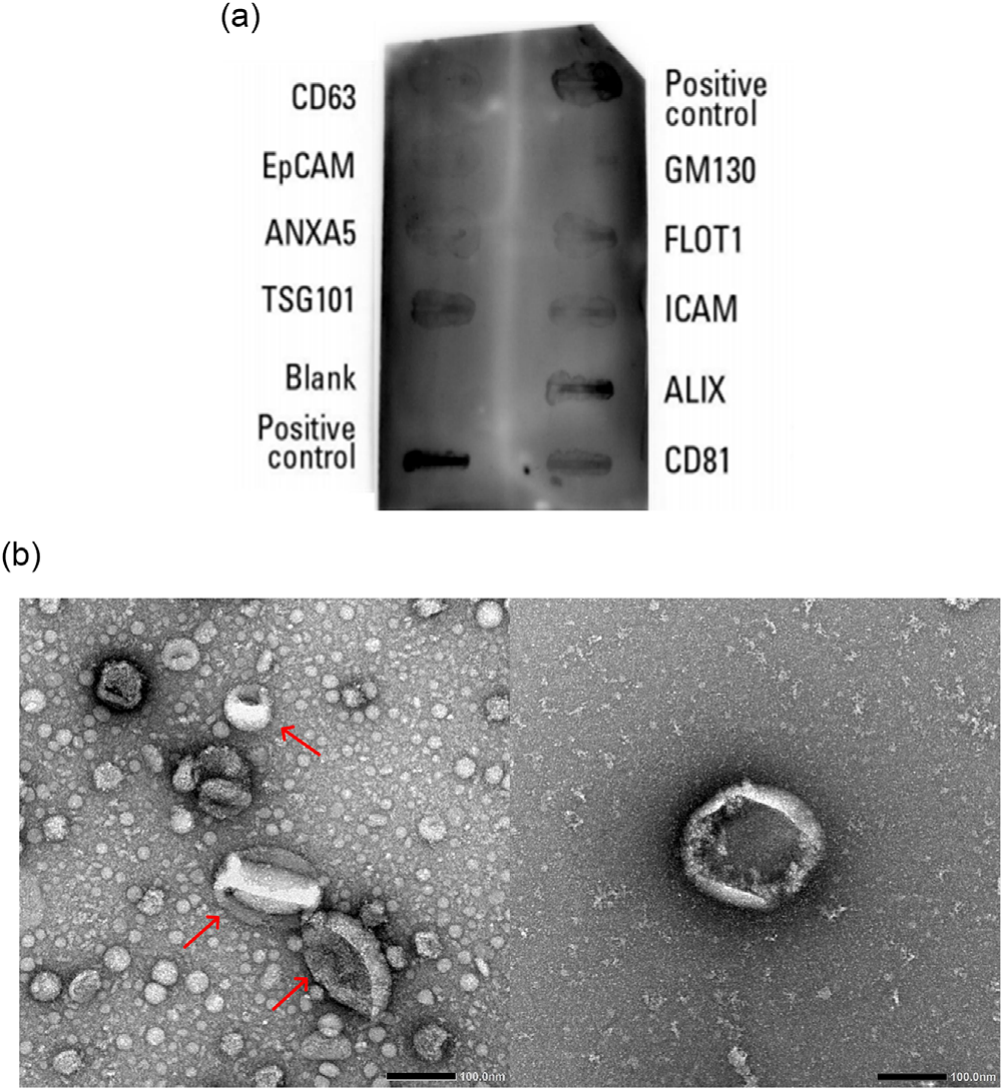
(a) Expression of standard EV protein markers in human milk EVs, as determined by Exo‐Check Array. Protein markers tested include FLOT1, ICAM, CD81, CD63, ANXA5 and TSG101. Positive control denotes labelled positive controls for horseradish peroxidase detection which indicates that the detection reagents were working properly, and the blank spot serves as a background control. (b) Morphology of human milk EVs, determined by transmission electron microscopy showing the structure. Left, a population of EVs (shown with red arrows); right, a single extracellular vesicle. Magnification 50,000×; Scale bar = 100 nm. ANXA5, Annexin A5; EV, extracellular vesicle; FLOT1, Flotillin 1; ICAM, Intercellular Adhesion Molecule‐1; TSG101, Tumour Susceptibility Gene 101.

Expression of EV protein markers, as identified by the Exo‐Check Array, is shown in Figure 1a. EVs are typically determined and characterized by using more than two positive antibodies against marker proteins, such as CD63, CD81 and ALG‐2‐interacting protein X (Alix) (Campos‐Silva et al., 2019; Leiferman et al., 2019; Levy et al., 2020; Matic et al., 2020; Théry et al., 2018). General markers of EVs, such as CD81, Alix and tumour susceptibility gene 101 protein (TSG101) were expressed in isolated EVs in this study, with Flotillin‐1 (FLOT1) and Intercellular Adhesion Molecule‐1 (ICAM) also detectable. Epithelial cell adhesion molecule (EpCAM), which is often found in cancer‐derived EVs, was also not apparent in the EVs. These findings are comparable to those in the literature (Bickmore & Miklavcic, 2020; Martin et al., 2018). For example, a previous study reported that human milk EVs were positive for CD81, ALIX, ICAM, TSG101 and Annexin A5, but negative for CD63 and EpCAM (Bickmore & Miklavcic, 2020). GM130, a Golgi apparatus protein which normally exists in eukaryotic cells but considered to be absent in EVs (Kalimuthu et al., 2018; Lötvall et al., 2014), was not expressed, which corroborated our proteomics results. In terms of the morphology of the isolated EVs, which was determined using transmission electron microscopy, the results (Figure 1b) revealed a typical ‘cup‐shaped’ (collapsed vesicle) appearance of EVs, which results from drying during sample preparation (Théry et al., 2006). These structures were ≥ 100 nm in diameter.

The term EVs is used here for the particles isolated and tested in this study since we did not examine their biogenesis. However, the data suggest that they are consistent with exosomes or small EVs based on the method of isolation, particle size, and protein expression.

### 3.2 Effect of simulated intestinal fluids and digestive enzymes on EVs

**FIGURE 2:**
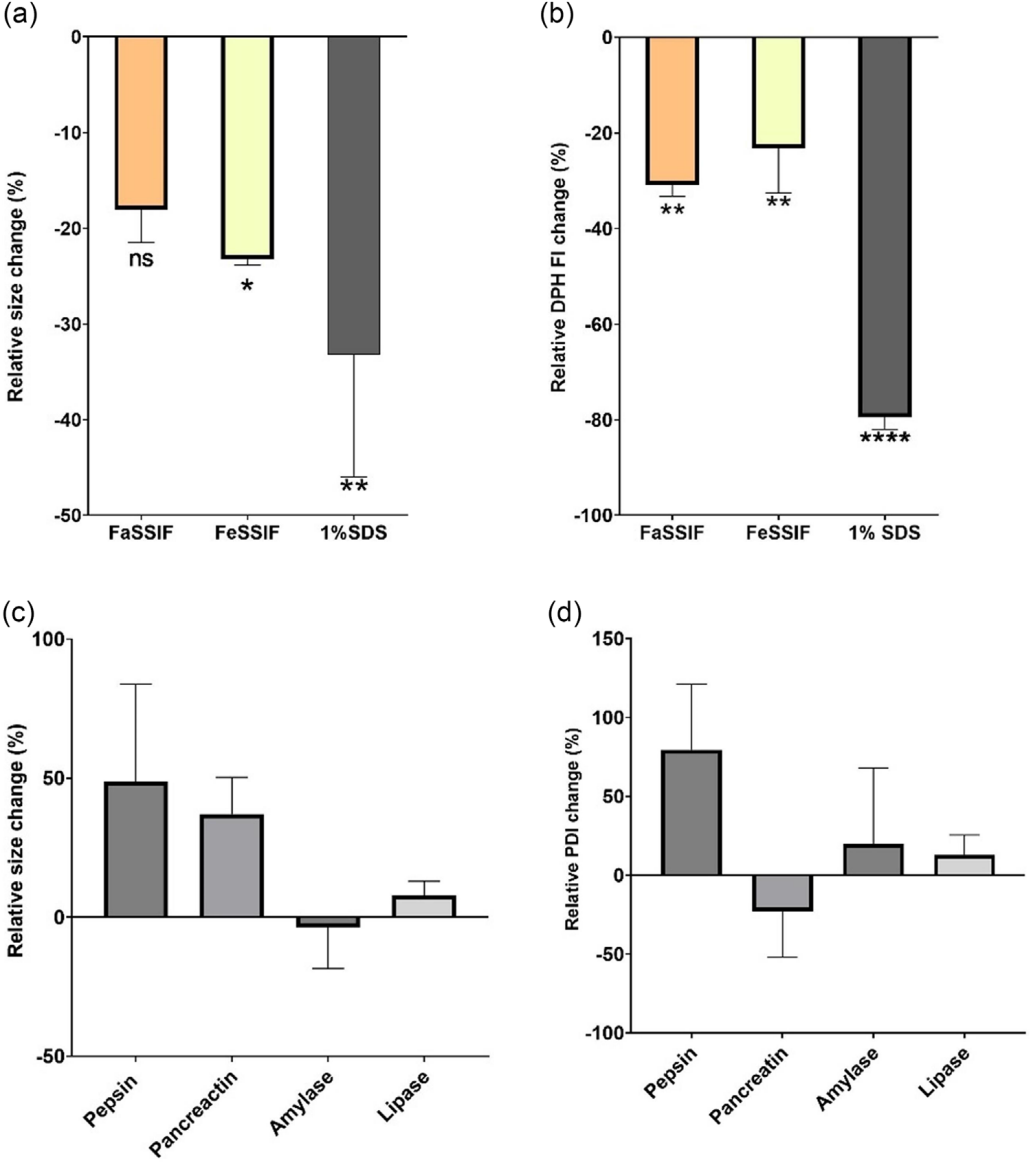
Effect of gut digestion on the stability (size) of human milk EVs. (a) Effect of FaSSIF and FeSSIF on EV particle size. The relative size change compared to EVs in PBS is shown. (b) Relative change in EV‐associated DPH fluorescence intensity. SDS was used as a control to solubilise EVs. Comparison relative to EVs in PBS. (C) Effect of digestion enzymes on EV particle size (*n* = 6). The size of EVs was measured before and after a 90 min digestion and the relative size change is shown. Pepsin was dissolved in 10 mM HCl solution at pH 1.5. Pancreatin was prepared in FaSSIF. Amylase and lipase were dissolved in deionised water. (d) Effect of digestion enzymes on PDI of EVs (*n* = 4). *, **, *** and **** indicate *p* < 0.05, *p* < 0.01, *p* < 0.001 and *p* < 0.0001, respectively. Results are mean ± SD (*n* = 3). DPH, 1,6‐Diphenyl‐1,3,5‐hexatriene; EV, extracellular vesicle; FaSSIF, Fasted State Simulated Intestinal Fluid; FeSSIF, Fed State Simulated Intestinal Fluid; PBS, phosphate‐buffered saline; PDI, polydispersity index; SDS, Sodium dodecyl sulphate.

The stability of human milk EVs was investigated by analysing the percentage change in the size of EVs and their membrane integrity in the presence of SSIFs. Figure 2a shows the size of EVs following exposure to SSIFs or controls. The results indicate that the treatment of EVs with FaSSIF did not have a significant impact on their size, with EVs displaying a statistically insignificant change in size to those in PBS. By contrast, FeSSIF and SDS treatment resulted in a decrease in the diameter of EVs.

To evaluate the effect of SSIF on EV membrane stability, DPH was utilised as a probe to indicate EV membrane intactness. EVs were exposed to SSIFs, or SDS and PBS controls. Figure 2b shows that compared with PBS controls, FaSSIF‐ and FeSSIF‐digested EVs exhibited a small but significant loss of DPH fluorescence, which amounted to 20%−30%, while SDS‐incubated EVs displayed a notably larger (79%) decrease in DPH fluorescence. The results therefore indicate that human milk EVs largely maintained membrane stability upon exposure to FaSSIF or FeSSIF. This is in line with previous findings showing that human milk EVs are resilient to digestion and their microRNAs (miRNA) survive digestion in vitro (Kahn et al., 2018; Liao et al., 2017). This is also in line with findings related to the stability of bovine milk EVs in intestinal biofluids (Zhang et al., 2023).

We also probed the effects of different digestive enzymes on the stability of EVs, specifically size. The change in size and PDI of EVs was measured after exposure to enzymes α‐amylase, pepsin, pancreatin and lipase (Figure 2c, d). After a 90 min exposure to these treatments, EVs displayed an increase in size following treatment with pepsin and pancreatin, while amylase and lipase did not influence their size.

### 3.3 Mucus diffusivity of human milk EVs

**FIGURE 3:**
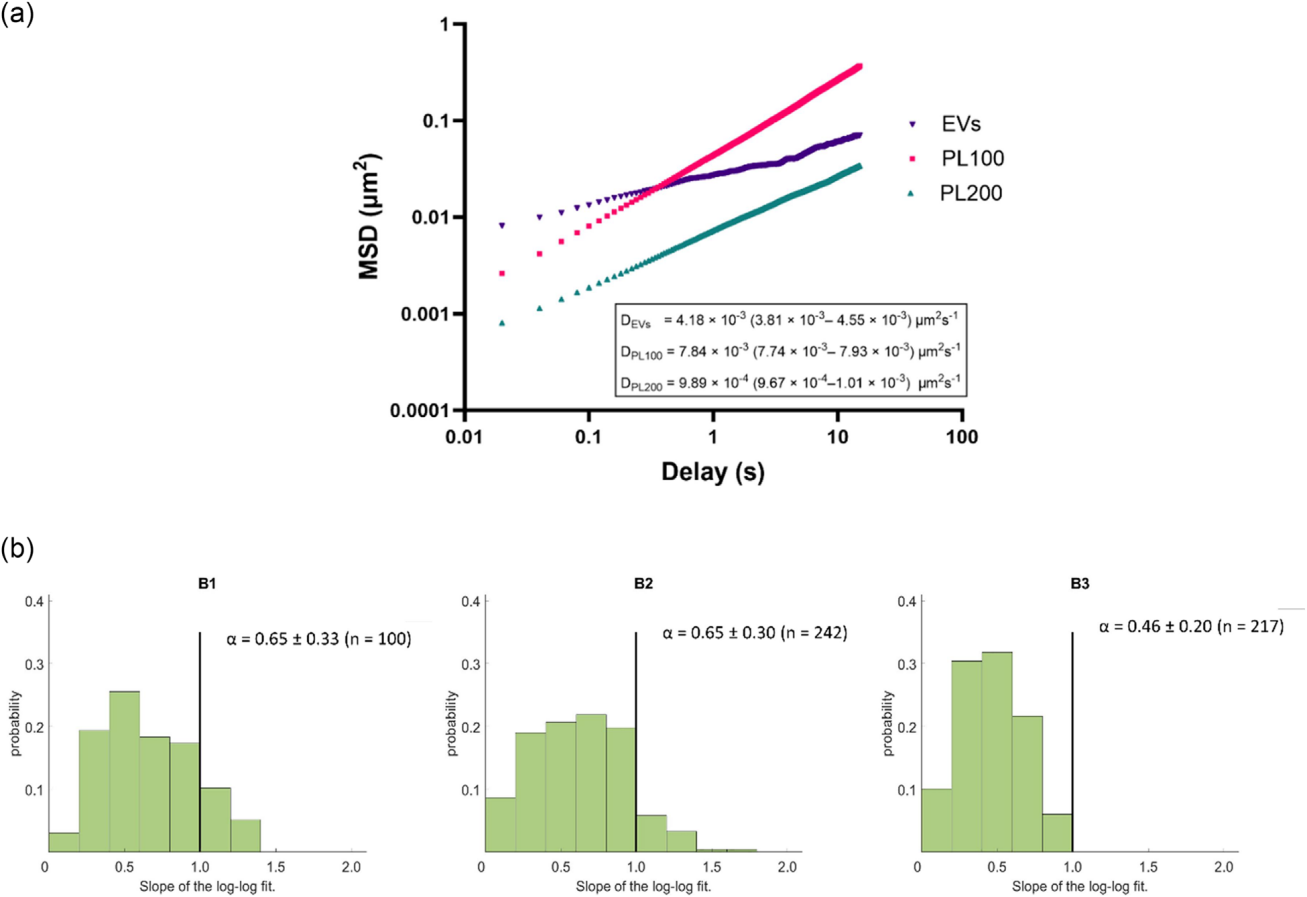
Mucus diffusion of human milk EVs and polystyrene latex nanoparticles. (a) Mean time‐weighted MSD values for analysed particle trajectories of EVs (*n* = 454), PL100 (*n* = 296), and PL200 (*n* = 278) diffusing in mucus, and calculated diffusion coefficient D. Diffusion coefficient values are shown with 95% confidence intervals. (b) Histogram showing the probability distribution of slopes of log‐log fit (alpha) values of particle trajectories of (B1) EVs, (B2) PL100 and (B3) PL200 nanoparticles. EV, extracellular vesicle.

Following analysis of the stability of human milk EVs in the presence of intestinal biofluids, we were interested in probing their diffusivity in mucus. This is important because there appears to be only one study (Warren et al., 2021) in this area. We employed particle tracking analysis for the first time to investigate the mucus diffusivity of human milk EVs. From the results, we were able to calculate the diffusion coefficient value of EVs, which was compared to negatively charged polystyrene nanoparticles 100 and 200 nm in diameter (PL100 and PL200, respectively). This comparison was made since negatively charged nanoparticles are known to possess significantly higher diffusivity in gastrointestinal mucus than positively charged nanoparticles, with a previous study showing that anionic polystyrene nanoparticles similar to those employed in this work diffused 20−30 times faster than cationic particles in gastrointestinal mucus (Crater & Carrier, 2010). The results showed that gastrointestinal mucus diffusion of EVs (4.18 × 10^−3^ µm^2^s‐^1^ fell in the range between that of PL100 (7.84 × 10^−3^ µm^2^s‐^1^) and PL200 (9.89 × 10^−3^ µm^2^s‐^1^) nanoparticles (Figure 3a). The plot of MSD(t) for the tested particles appeared to be linear for low values of delay, suggesting unconstrained particle movement at short time intervals. The mean values of the slope of the log‐log fit were assessed to determine whether the movement of EVs in mucus remains unconstrained at longer values of delay. No significant differences were observed between mean slopes (alpha) of log‐log fit values in EVs or PL100 nanoparticles, with mean slope values of around 0.65 indicating primarily diffusive motion with some impediment at longer time scales (Figure 3b) (Qian et al., 1991). Lower PL200 nanoparticle alpha value of 0.46 is indicative of increasingly constrained particle motion with an increase in particle size to 200 nm.

### 3.4 Cellular uptake and intestinal transport of EVs

**FIGURE 4:**
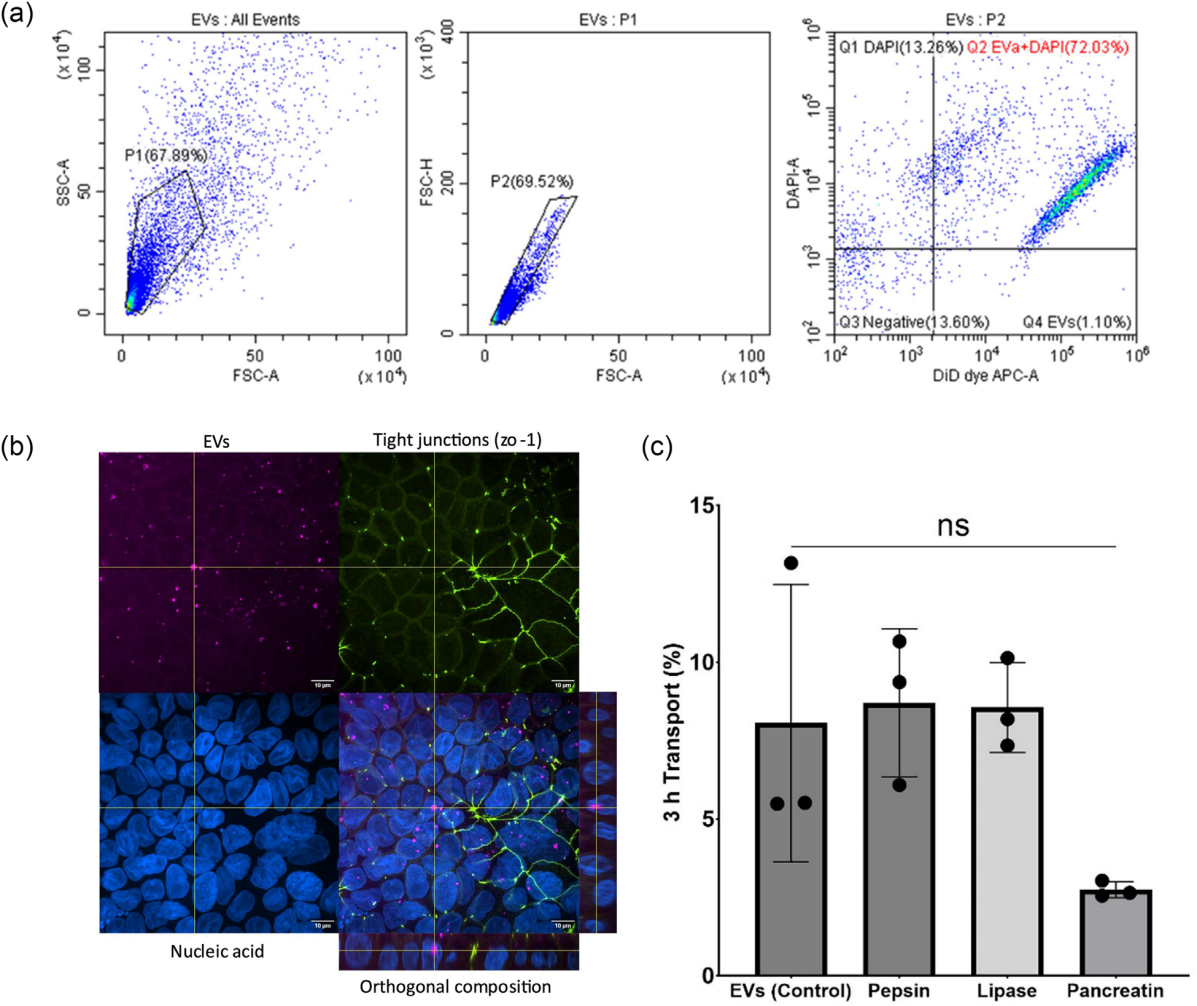
Intestinal cellular uptake and transport of human milk EVs. (a) Representative flow cytometry scatter plots of Caco‐2 cells representing the cellular uptake of EVs. The right double scatter plot shows side scattering versus forward scattering of Caco‐2 cells. The middle double scatter plot is based on peak height versus peak area of forward scattering. The left plot shows the intensity of DAPI versus DiD dye or FITC dye, which indicates the percentage of cells with positive signals for DAPI and EVs (red). (*n* = 3). B) Confocal imaging of cellular uptake of EVs (red) in differentiated Caco‐2 cells. Cell nuclei are stained with DAPI (blue) and tight junctions are stained using a ZO‐1 tight junction protein antibody (green). Scale bar = 10 µm. C) Transport of EVs across intestinal epithelial Caco‐2 monolayers following a 3 h application, with or without exposure to different digestive enzymes (pepsin, lipase and pancreatin). EVs were stained with DiD dye and untreated EVs served as controls (*n* = 3). *, ** and **** indicate *p* < 0.05, *p* < 0.01 and *p* < 0.0001, respectively. EV, extracellular vesicle.

Following a 3 h treatment of intestinal epithelial Caco‐2 cells with fluorescently labelled EVs, 68.46% ± 7.32% of Caco‐2 cells contained EV fluorescence signals (*n* = 6), as determined using flow cytometry. Representative plots (Figure 4a) show the percentage of cells with positive fluorescence signals following treatment with human milk EVs. To enable this analysis, cell debris and doublets were excluded using appropriate gates. A positive DID dye signal indicates cells internalising EVs, while a DAPI‐associated signal indicates live cells.

Figure 4b shows that EV fluorescence signals (red) were clearly associated with the cells and located throughout the cytosol.

In terms of intestinal epithelial translocation of EVs, Figures 4c and S1 (Supplementary Information) show that EVs displayed a significant permeation (∼8% and ∼18%, respectively of the total applied in 3 h) across human intestinal Caco‐2 monolayers. EVs treated with pepsin, lipase or pancreatin enzymes displayed similar epithelial transport to untreated EVs, although pancreatin‐exposed EVs showed a statistically insignificant decrease in translocation (Figure 4c).

### 3.5 Effect of endocytosis inhibitors on intestinal uptake and permeation of EVs

**FIGURE 5:**
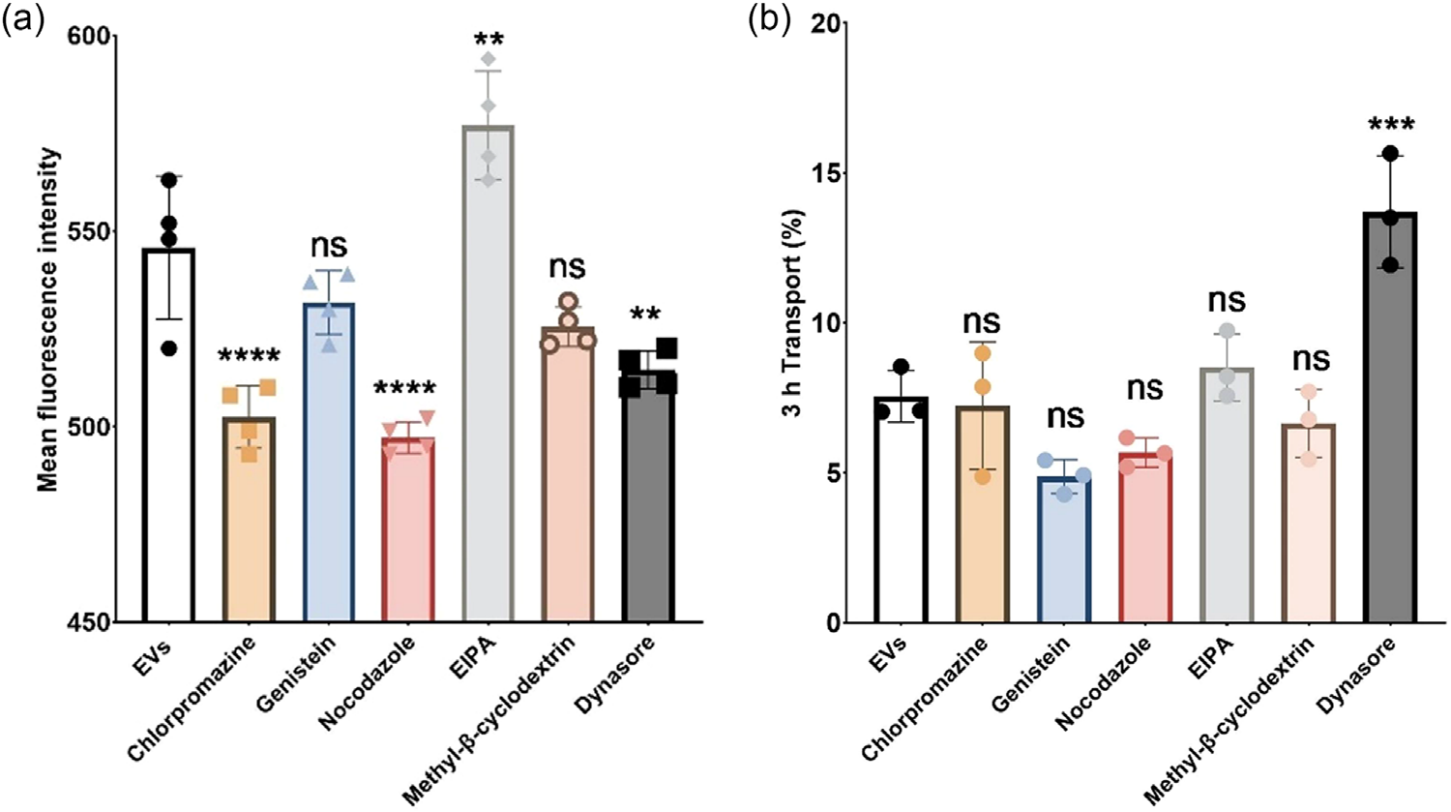
Effects of pharmacological inhibitors of endocytosis pathways on the internalization and transport of human milk EVs in intestinal epithelial Caco‐2 monolayers. (a) Internalization of EVs in Caco‐2 monolayers treated with different endocytosis inhibitors, determined via flow cytometry. The monolayers were pretreated with inhibitors for 30 min and EVs were applied to cells at 0.05 mg/mL for 3 h. (b) Transport of EVs with different endocytosis inhibitors. ** and **** indicate *p* < 0.01 and *p* < 0.0001, respectively. EV, extracellular vesicle.

To shed light on the possible mechanism(s) of intestinal uptake and transport of EVs, we utilised pharmacological cell uptake inhibitors and determined their impact on these phenomena in Caco‐2 monolayers. The inhibitors were used at concentrations that were previously shown to exert no cytotoxicity towards Caco‐2 cells (Tinevez et al., 2017) and included chlorpromazine which inhibits clathrin‐dependent endocytosis (Crater & Carrier, 2010), and nocodazole as an inhibitor of macropinocytosis. The results showed that both chlorpromazine and nocodazole induced a significant reduction in EV uptake in Caco‐2 cells (Figure 5a). Genistein, a tyrosine kinase inhibitor that blocks the caveolae‐mediated uptake pathway (Crater & Carrier, 2010), displayed no significant effect on the cellular uptake of EVs. The application of EIPA, an inhibitor of macropinocytosis, resulted in an increase in the cell uptake of EVs. Methyl‐β‐cyclodextrin, which depletes cholesterol from cell membranes (Mahammad & Parmryd, 2008), did not have an impact on the cell uptake of EVs (Figure 5a). Finally, the co‐application of EVs with the caveolae inhibitor dynasore resulted in a decrease in cell uptake of EVs. With the exception of dynasore which, for reasons that are presently unclear, increased the epithelial transport of EVs, none of the tested inhibitors had an effect on the Caco‐2 transport of human milk EVs (Figure 5b).

### 3.6 Proteomic analysis of EVs

**FIGURE 6:**
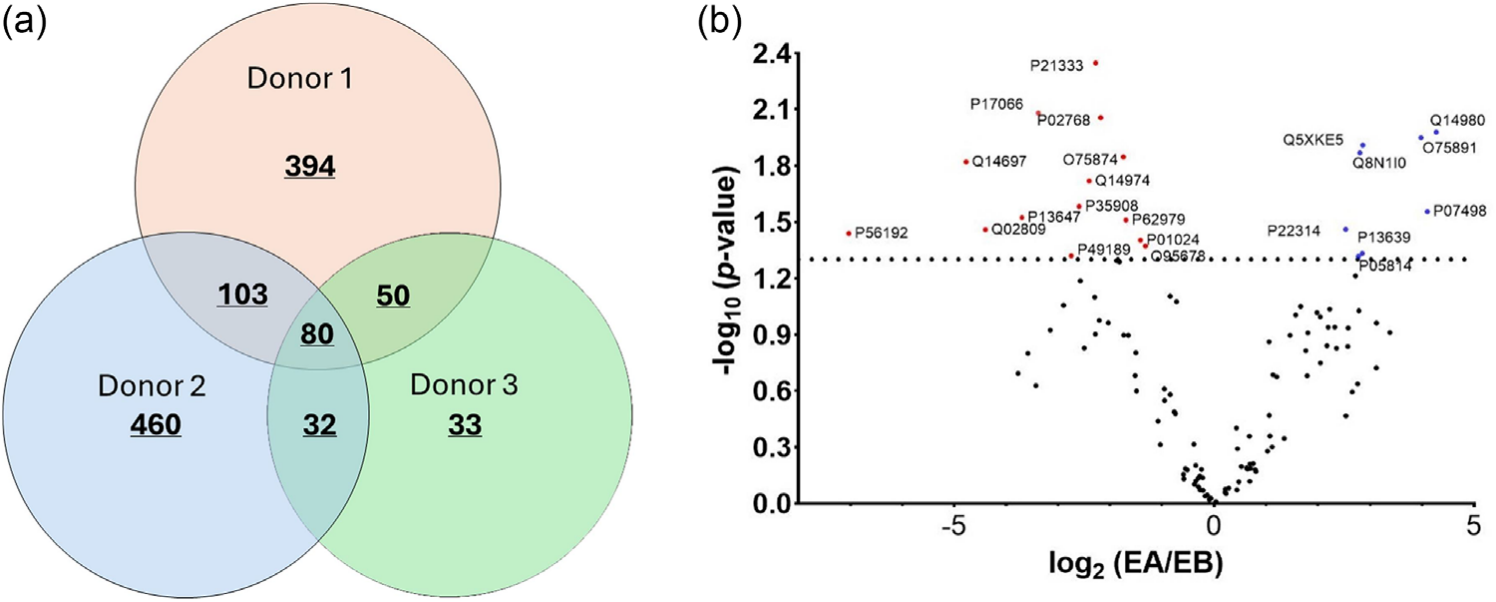
(a) Venn diagram showing common proteins of human milk EVs from three different donors. Eighty proteins are present in all three groups. (b) Volcano plot showing human milk EV proteins in terms of apical sides (EA) and basolateral sides (EB). EV proteins significantly more or less abundant on the basolateral side are indicated in red and blue, respectively, with a *p* < 0.05 (*n* = 5). Proteins identified in at least 3 out of 5 replicates (or above 70%) were considered as positive identification. EV, extracellular vesicle.

Proteomic analysis of human milk EVs from three different donors yielded different total numbers of proteins (627, 675 and 195; Figure 6a). Eighty of the identified proteins were common to all three donors and a further 153 were common to two out of three donors. A total of 1152 proteins were identified in EVs from the three donors.

A total of 22 proteins exhibited differences in enrichment in EVs transported across intestinal epithelial cells (remaining on the apical side of Caco‐2 monolayers) and transported EVs sampled from the basolateral side. In Figure 6b, proteins with higher concentrations on the basolateral side are highlighted in red and those with a lower concentration are coloured blue. Proteomic analysis revealed 14 proteins that are enriched in transported EVs versus non‐transported counterparts (adjusted *p* < 0.05). These 14 proteins are listed in Table 2, alongside statistical analysis. On the other hand, the EV proteins which are diminished at the basolateral side are listed in Supplementary Information, Table S1. It must be noted that keratins were not removed from the result as contaminations since they may be produced from mammary epithelial cells which generate EVs (van Herwijnen et al., 2016). PPI and gene ontology (GO) analysis are shown in Supplementary Information (Figures S2 and S3, respectively).

**TABLE 2:** List of 14 EVs proteins with significant differences between apical (EA) and basolateral (EB) chambers (EA < EB, *p* < 0.05). EA/EB is the ratio of EV abundance in apical versus basolateral chambers. Proteins are ranked by *p*‐value (*n* = 5).

UniProt ID	Protein name	Gene name	Log_2_(EA/EB)	Student's *t*‐test *p*‐value
P21333	Filamin‐A	FLNA	−2.275	0.004
P17066	Heat shock 70 kDa protein 6	HSPA6	−3.381	0.008
P02768	Serum albumin	ALB	−2.181	0.009
O75874	Isocitrate dehydrogenase 1	IDH1	−1.746	0.014
Q14697	Neutral alpha‐glucosidase AB	GANAB	−4.773	0.015
Q14974	Importin subunit beta‐1	KPNB1	−2.401	0.019
P35908	Keratin, type II cytoskeletal 2 epidermal	KRT2	−2.593	0.026
P13647	Keratin, type II cytoskeletal 5	KRT5	−3.698	0.030
P62979	Ubiquitin‐40S ribosomal protein S27a	RPS27A	−1.697	0.031
Q02809	Procollagen‐lysine,2‐oxoglutarate 5‐dioxygenase 1	PLOD1	−4.403	0.035
P56192	Methionine–tRNA ligase, cytoplasmic	MARS1	−7.030	0.036
P01024	Complement C3c alpha' chain fragment 1	C3	−1.416	0.040
O95678	Keratin, type II cytoskeletal 75	KRT75	−1.320	0.042
P49189	4‐trimethylaminobutyraldehyde dehydrogenase	ALDH9A1	−2.747	0.048

## 4 DISCUSSION

Human milk EVs may play a crucial role as carriers of biological signals from mother to baby (Jiang et al., 2021; Kupsco et al., 2021). To be able to transfer biological signals in the form of biological molecules, such as miRNAs, from mother to baby, these EVs must both protect the biomolecule cargo in the harsh biochemical environment of the gut and transport it to the cells of the gastrointestinal tract and/or beyond. Indeed, there is now clear evidence that human milk EVs are able to survive digestion and transport across the human intestinal epithelium (Jiang et al., 2021). There is a need to study the biological mechanisms of resistance to digestion and intestinal epithelial transport of human milk EVs for multiple reasons. First, there may be scope for the therapeutic use of human milk EVs since they have been reported to demonstrate biological effects that may potentially be harnessed therapeutically. For example, human milk EVs have been shown to promote intestinal epithelial cell proliferation (Dong et al., 2020; Pisano et al., 2020; Wang et al., 2022), protect against necrotising enterocolitis (NEC) (Chen et al., 2021; Pisano et al., 2020; Wang et al., 2019), promote the expression of proteins mediating intestinal barrier function (He et al., 2021), and enhance epithelial barrier function (Tong et al., 2023; Zonneveld et al., 2021). Understanding gut stability and intestinal transport of human milk EVs may facilitate the potential development of such therapies. Second, studying the interactions of human milk EVs with the biological barriers of the gut could also inform the field of drug delivery. To this end, there is a major need for drug delivery systems that possess the properties of human milk EVs in terms of their behaviour in the gut to enable oral delivery of biological therapies. We were therefore interested in gaining more insight into the interactions of human milk EVs with the biochemical and biophysical barriers of the gut and deciphering the intestinal epithelial transport processes and potential EV proteins involved in the gut uptake of human milk EVs.

EVs isolated and analysed in this work displayed similar physicochemical characteristics (Table 1) to those reported previously (Bickmore & Miklavcic, 2020). EVs also expressed established EV markers and displayed an expected and previously reported morphology (Figure 1). Stability studies indicate that human milk EVs are not markedly affected upon exposure to FaSSIF or FeSSIF. While there we observed a decrease in the diameter of EVs (Figure 2a) following treatment with FeSSIF, together with changes in DPH fluorescence (Figure 2b), these changes in general were lower than those resulting from SDS treatment. To test the membrane stability of EVs, we employed a fluorescence‐based assay based on the DPH probe to indicate EV membrane intactness. DPH, which is conventionally used to determine membrane fluidity, is weakly fluorescent in water and its fluorescence is drastically increased on formation of supramolecular assemblies with lipids within a hydrophobic environment (Caudron et al., 2007), such as EV membranes. For this reason, we previously successfully validated and employed this probe to test the membrane stability of EVs (Zhang et al., 2024), based on the postulation that EV membrane disturbance would result in DPH release into water and a measurable decrease in fluorescence signal. Our stability studies are in line with previous findings showing that human milk EVs are resilient to digestion and their miRNAs survive digestion in vitro (Kahn et al., 2018; Liao et al., 2017). This is also in line with findings related to the stability of bovine milk EVs in intestinal biofluids (Zhang et al., 2023). In terms of the effects of different digestive enzymes on human milk EV size, treatment with pepsin and pancreatin induced a change (increase) in EV size, while amylase and lipase did not have an effect (Figure 2). Again, these observations are in agreement with the literature reporting that human milk EVs are minimally impacted by exposure to several gastrointestinal proteases (Kahn et al., 2018; Kosaka et al., 2010; Liao et al., 2017).

Mucus diffusivity analysis confirmed that human milk EVs possess a similar ability to diffuse in gastrointestinal mucus to nanoparticle systems of similar size that are known to have high mucus diffusivity (Figure 3). These findings, which are novel with respect to human milk EVs, therefore point to their ability to readily diffuse across this important physiological barrier in the gastrointestinal tract. These observations are comparable to those reported for bovine milk EVs (Warren et al., 2021).

Cell uptake studies of human milk EVs, performed using flow cytometry, revealed that 69.5% of intestinal epithelial Caco‐2 cells were associated with EV fluorescence signals. Cell uptake of EVs was also confirmed using confocal imaging (Figure 4). Regarding intestinal epithelial transport, human milk EVs were associated with a significant level of translocation (∼8% of the total applied) in human intestinal Caco‐2 monolayers. Interestingly, treatment of EVs with gut enzymes pepsin, lipase or pancreatin did not have an impact on the extent of epithelial translocation of EVs. This, together with the findings that SSIFs did not affect the size of EVs, suggests that human milk EVs survive the digestion conditions, and the capacity for these EVs to translocate across the intestinal epithelium is maintained upon exposure to a digestive environment. These observations mirror those reported in a recent study by Yung et al. showing that human milk EVs survive human neonatal digestion and are rapidly absorbed ex vivo by neonatal enteroids  (Yung et al., 2024).

Endocytosis pathways that may be involved in epithelial cell uptake and transport of human milk EVs were investigated using pharmacological inhibitors, and several of the inhibitors had an impact on the cell uptake, with chlorpromazine, nocodazole and dynasore decreasing the uptake of EVs, while EIPA increased cell uptake in intestinal epithelial cells (Figure 5a). Chlorpromazine, an inhibitor of clathrin‐dependent endocytosis, and nocodazole, an inhibitor of micropinocytosis, had the most prominent effects on EV uptake, while inhibition of the caveolae‐mediated uptake pathway by genistein (Crater & Carrier, 2010) and cell membrane cholesterol depletion by methyl‐β‐cyclodextrin (Mahammad & Parmryd, 2008) did not influence the cellular uptake of EVs. For reasons that are currently not clear, EIPA, an inhibitor of macropinocytosis, increased cell uptake of EVs. The effects of pharmacological inhibitors of specific cell uptake pathways therefore suggest that human milk EVs are taken up into intestinal epithelial cells via multiple pathways. Interestingly, however, the epithelial translocation of EVs was not affected by the pharmacological inhibitors, with the exception of dynasore (caveolae inhibitor) which, for reasons that are presently unclear, increased the epithelial transport of EVs.

Studies examining the mechanisms of intestinal epithelial cell uptake of human milk EVs are limited. However, a recent seminal study, which carefully replicated the in vivo conditions of human milk EV interactions with the infant's gut by isolating EVs from infant intestinal contents post‐feeding and testing their uptake in neonatal enteroids (organoids), reported that such EVs are rapidly taken up by neonatal enteroids through a process that partially depends on dynamin‐mediated endocytosis (Yung et al., 2024). Our results are consistent with this study because the use of the same pharmacological inhibitor (dynasore) decreased the uptake of human milk EVs in Caco‐2 cells.

While the cellular uptake mechanisms of human milk EVs have not been studied extensively, other types of EVs have been investigated in this regard, and this topic has been reviewed in depth recently (Gandek et al., 2023). The conclusion from these studies is that the intracellular trafficking pathway of EVs generally follows the endo‐lysosomal pathway, with multiple uptake mechanisms contributing to EV uptake, although seemingly all leading to accumulation in lysosomes (Gandek et al., 2023).

Proteomic studies of human milk EVs have been reported in the literature, though these studies focussed on compositional analysis and identification of the functional proteome, rather than identification of candidate proteins that may facilitate intestinal epithelial translocation. Our LC‐MS analysis identified 1152 proteins across all three donors combined (627, 675 and 195 in donors 1, 2 and 3, respectively). The discrepancy in the number of identified proteins between the donors is unclear and could be attributed to a number of factors, including variations in particle size and number (and protein mass) of the analysed samples, together with variations between donors such as the stage of lactation. Such a variation has been observed in previous proteomic studies, with the number of identified proteins for low‐density human milk EVs reported to be between 149 and 1615 based on the size of EVs (van Herwijnen et al., 2016). A limitation of our study is the use of a small number of biological replicates, which makes it challenging to determine the true physiological variability in protein expression profile of human milk EVs. Fourteen proteins were found to be enriched in EVs transported across the intestinal epithelial monolayers (Table 2). According to GO, all 14 proteins are found in EVs, with 13 of these expressed in endocrine glands and the female reproductive system and 10 proteins found in the liver. Out of these 14 proteins, we identified EV membrane proteins Filamin‐A, C3 and Heat shock 70 kDa protein 6.

Overall, this work provides new information related to the interactions of human milk EVs with biochemical and biophysical components of the gastrointestinal tract, including diffusion of these particles in gastrointestinal mucus, endocytic pathways of cell uptake, and EV proteins that may facilitate intestinal epithelial translocation. The work strengthens the existing evidence that EVs in human milk may be important signalling mediators based on their ability to carry biological cargo into and across intestinal epithelial cells within the hostile biochemical environment of the gut. Future work should focus on studying the functionality of biological cargo of these EVs in recipient cells within the gut or systemically, together with associated roles in communication between mother and child.

## AUTHOR CONTRIBUTIONS

**Xiang Luo**: Formal analysis (lead); funding acquisition (lead); investigatio (lead); writing–original draft (supporting). **Yunyue Zhang**: Investigation (supporting); methodology (supporting); supervision (supporting). **Ning Ding**: Investigation (supporting). **Jana Javorovic**: Investigation (supporting). **Bahijja Tolulope Raimi‐Abraham**: Supervision (supporting); writing–original draft (supporting). **Steven Lynham**: Data curation; formal analysis; investigation. **Xiaoping Yang**: Data curation (equal); formal analysis (equal); investigation (equal). **Natalie Shenker**: Resources (supporting); supervision (supporting); writing–original draft (supporting). **Driton Vllasaliu**: Conceptualization (lead); investigation (supporting) (lead); methodology (lead); project administration (lead); supervision (lead); visualization (lead); writing–original draft (lead); writing–review and editing (lead).

## CONFLICT OF INTEREST STATEMENT

N.S. is the co‐founder of the Human Milk Foundation, a charity dedicated to providing equitable access to donor human milk in the UK, which operates the Hearts Milk Bank.

## Supporting information



Supplementary information

## Data Availability

The mass spectrometry proteomics data have been deposited to the ProteomeXchange Consortium via the PRIDE (Perez‐Riverol et al., 2022) partner repository with the dataset identifier PXD057096. We have submitted all relevant experimental parameters to the EV‐TRACK knowledgebase (EV‐TRACK ID: EV240191) (Van Deun et al., 2017).
