# Supplementary material for: Mechanistic insight into human milk extracellular vesicle‐intestinal barrier interactions
Source: J Extracell Biol. 2025 Jan 9;4(1):e70032. doi: 10.1002/jex2.70032 (PMC11714171; doi:10.1002/jex2.70032)
Supplement: Supplementary file 1 — Supplementary information [file JEX2-4-e70032-s001.docx]

**Supplementary Information**


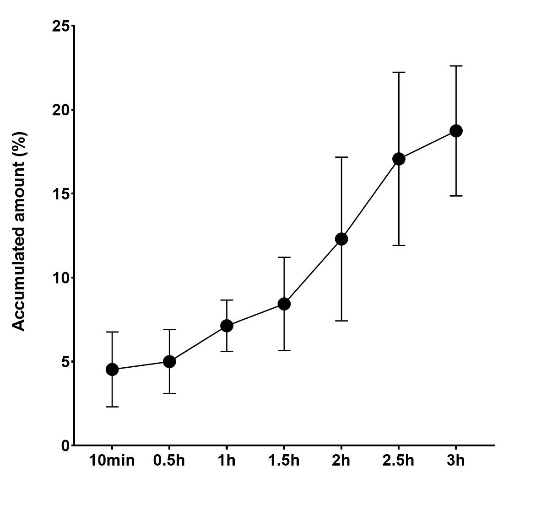


**Figure S1. *Transport of human milk EVs across Caco-2 intestinal epithelial monolayers.*** *Percentage of EVs accumulated on the basolateral side of Transwell-cultured Caco-2 monolayers is shown at different time intervals, from 10 min to 3 h (n=3).*


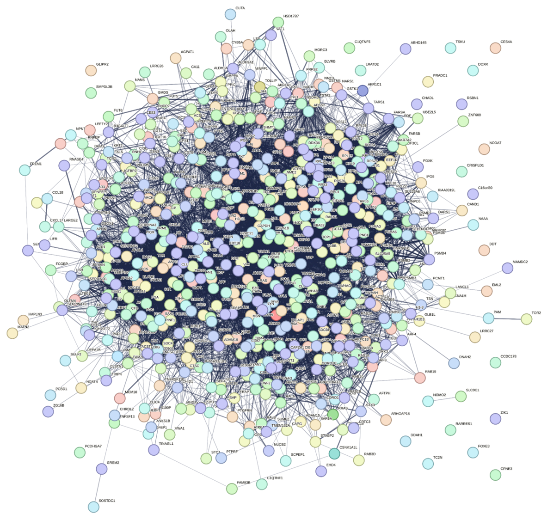


**Figure S2.** ***Protein-protein interaction (PPI) network representing the interactions between proteins of hmEVs using STRING****. Nodes and edges are human milk EV proteins and physical or functional interactions, respectively. Interactions with medium or high confidence are shown and the thickness of edges reflects the strength of the confidence level.*

**Figure S3 Percentage and the p value of gene associated with transportation in GO term**. The gene set was annotated with biological process. The gene were ranked by enriched percentage. The p value of all terms was smaller than 0.05 which indicates the enriched gene associated with transportation were significant. The data were analysed by STRING.

**Table S1. List of 8 EV proteins with significant differences between apical (EA) and basolateral (EB) sides of Caco-2 intestinal epithelial monolayers following transport study, where EA is higher than EB (EA > EB, p < 0.05).** EA/EB is the ratio of EV abundance in apical vs. basolateral chambers. Proteins are ranked by p-value (n = 5).

| UniProt ID | Protein name | Gene name | Log_2_(EA/EB) | Student's T-test *p* -value |
| --- | --- | --- | --- | --- |
| Q14980 | Nuclear mitotic apparatus protein 1 | NUMA1 | 4.268393 | 0.010527 |
| O75891 | Cytosolic 10-formyltetrahydrofolate dehydrogenase | ALDH1L1 | 3.976899 | 0.011244 |
| Q5XKE5 | Keratin, type II cytoskeletal 79 | KRT79 | 2.858522 | 0.012351 |
| Q8N1I0 | *Dedicator of cytokinesis protein 4* | DOCK4 | 2.801479 | 0.013564 |
| P07498 | Kappa-casein | CSN3 | 4.099499 | 0.027832 |
| P13639 | *Elongation factor 2* | EEF2 | 2.529645 | 0.034541 |
| P22314 | *Ubiquitin-like modifier-activating enzyme 1;* | UBA1 | 2.849599 | 0.046399 |
| P05814 | *Beta-casein* | CSN2 | 2.771528 | 0.048124 |
